# Peculiarities of hyperlipidaemia in tumour patients.

**DOI:** 10.1038/bjc.1981.94

**Published:** 1981-05

**Authors:** V. M. Dilman, L. M. Berstein, M. N. Ostroumova, Y. V. Tsyrlina, A. G. Golubev

## Abstract

The study group included 684 cases: 258 patients with breast carcinoma, 113 males with lung cancer, 42 patients with rectal tumours, 42 patients with stomach tumours, 59 patients with fibroadenomatosis, and 170 healthy subjects of varying age (male and female). A relatively high blood triglyceride level was found in patients with breast, lung, rectal (females), and stomach (female) tumours. The blood concentration of high-density lipoprotein-cholesterol in patients with breast, lung, and stomach (female) tumours was relatively low. The elimination of tumour (breast carcinoma) did not lead to significant changes in lipid metabolism. There was no correlation between degree of lipidaemia and stage of tumour progression except in the cases of rectal cancer. Preliminary results are presented on the tentative classification of hyperlipoproteinaemia in tumour patients, using the lipid concentration threshold values advocated by Carlson et al. (1977); an increased frequency of Type IV hyperlipoproteinaemia proved to be the most characteristic feature of tumour patients. The results are discussed in terms of the concept of the importance of lipid metabolic disturbances, primarily those due to ageing, in the genesis of the syndrome of "cancerophilia" (predisposition to cancer).


Br. J. Cancer (1981) 43, 637

### PECULIARITIES OF HYPERLIPIDAEMIA IN TUMOUR PATIENTS

V. M. DILMAN, L. M. BERSTEIN, M. N. OSTROUMOVA, Y. V. TSYRLINA AND A. G. GOLUBEV

*From the Laboratory of Endocrinology, Petrov Research Institute of Oncology, Leningrad, USSR*

Received 16 June 1980 Accepted 27 January 1981

**Summary.—The study group included 684 cases: 258 patients with breast carcinoma, 113 males with lung cancer, 42 patients with rectal tumours, 42 patients with stomach tumours, 59 patients with fibroadenomatosis, and 170 healthy subjects of varying age (male and female).**

**A relatively high blood triglyceride level was found in patients with breast, lung, rectal (females), and stomach (female) tumours. The blood concentration of high-density lipoprotein-cholesterol in patients with breast, lung, and stomach (female) tumours was relatively low. The elimination of tumour (breast carcinoma) did not lead to significant changes in lipid metabolism. There was no correlation between degree of lipidaemia and stage of tumour progression except in the cases of rectal cancer.**

**Preliminary results are presented on the tentative classification of hyperlipoproteinaemia in tumour patients, using the lipid concentration threshold values advocated by Carlson *et al.* (1977); an increased frequency of Type IV hyperlipoproteinaemia proved to be the most characteristic feature of tumour patients. The results are discussed in terms of the concept of the importance of lipid metabolic disturbances, primarily those due to ageing, in the genesis of the syndrome of "cancerophilia" (predisposition to cancer).**

DISTURBANCES OF LIPID METABOLISM are a common feature of cancer patients (Begg, 1958). Proper evaluation of these disturbances is of major importance for elucidation of their origin, for the appraisal of their effects on the course of the tumour process, and finally for making decisions as to the advisability and type of corrective measures to be taken.

There are 2 principal approaches to the problem of the origin of metabolic disturbances in tumour patients. On the one hand, these disturbances may be the result of intensified transport of lipids and carbohydrates to the tumour caused by the influence of the tumour itself (Begg, 1958; Kavetsky, 1962; Shapot, 1975). Although this mechanism may well operate, especially in the later stages of tumour progression, a number of data and considerations point to other possible reasons for disturbances of lipid metabolism, such as nonspecific changes in the energy homoeostat which are not dependent upon the presence of a tumour and occur in the course of normal ageing, especially in some age-related diseases (Dilman, 1968, 1978*a*, 1979; Dilman *et al.*, 1979). These changes may be risk-factors for tumour diseases and may be controllable.

Since it is known that the blood lipids are components of circulating lipoproteins* which transport lipids (Eisenberg & Levy, 1975), the lipid metabolism of an

Authors' address: Laboratory of Endocrinology, Petrov Research Institute of Oncology, 68 Leningradskaya St, Pesochny-2, Leningrad 188646, U.S.S.R.

* Three main classes of lipoproteins are distinguished: lipoproteins of high, low, and very low density, designated HDL, LDL, and VLDL respectively.

organism should be studied and discussed in terms of lipoproteins. This was done in detail by Barclay *et al.* (1970) and Barclay & Skipsky (1975). Their finding that the level of HDL is notably decreased in tumour patients, and also in healthy subjects from families with a positive cancer history, lends support to the validity of such an approach. But classical methods of lipoprotein analysis require prolonged ultracentrifugation, are time-consuming and not available for most clinical laboratories. Therefore it seems important to evaluate some simplified approaches based on metal-polyanion precipitation (Burstein & Scholnick, 1973) and on calculations using data on total triglyceride and cholesterol content in blood. In contrast to studies of hypercholesterolaemia in cancer patients (*e.g.* Dilman & Bobrov, 1966; Feldman & Carter, 1971; Smethurst *et al.*, 1975), disturbances of triglyceride content in the blood of tumour patients have not received sufficient attention. Furthermore, there are no published data on attempts to type hyperlipoproteinaemia in tumour patients.

The aim of this paper is to present data on the levels of triglycerides, cholesterol and HDL-cholesterol in the blood of patients with breast, lung, stomach, and colonic tumours. An attempt will be made to specify the hyperlipoproteinaemia characteristic of oncological patients.

Some data on lipidaemia in patients with different neoplasms, mainly on the level of cholesterol and free fatty acids in blood have been published in earlier reports from this laboratory (Dilman & Bobrov, 1966; Dilman *et al.*, 1968; Tsyrlina *et al.*, 1977; Berstein *et al.*, 1978).

### MATERIALS AND METHODS

A total of 684 patients were examined: 258 breast-cancer patients aged 28–69; 113 males with lung cancer (aged 31–70); 42 cases of rectal cancer (18 males and 24 females, aged 37–69); 42 cases of stomach carcinoma (22 males and 20 females, aged 32–68); 59 patients with breast fibroadenomas (aged 23–69) and 170 healthy subjects (90 aged 20–29—45 male and 45 female, and 80 aged >45—35 male and 45 female). The group of breast-cancer patients included 185 cases of primary tumour and 73 in clinical remission (radical mastectomy followed by radiation and/or cytostatic drugs, not less than 12 months before examination). For the other sites, only patients with primary tumours were examined. Most of the tumour patients were in Clinical Stages II (a, b) and III (a, b), *i.e.* without clinical manifestations of distant metastasis. Blood cholesterol was assayed by a modification of the Lieberman–Burchardt reaction (King, 1947), triglycerides were estimated according to the method of Carlson (1963), total $\beta$-lipoproteins (VLDL + LDL) according to that of Ledvina & Coufalova (1960) and HDL-cholesterol was measured after removal of VLDL and LDL from the serum by precipitation with heparin and manganese chloride (Burstein & Scholnick, 1973). Threshold values of lipid concentrations for typing hyperlipoproteinaemia were chosen according to the recommendations of Carlson *et al.* (1977). Cases with cholesterol levels >290 mg/100 ml and triglycerides <180 mg/100 ml were referred to as Type IIa; cholesterol >290 mg/100 ml and triglycerides >180 mg/100 ml—Type IIb, and cholesterol <290 mg/100 ml and triglycerides >180 mg/100 ml—Type IV. The blood level of LDL-cholesterol (LDL-Ch) was obtained from the relation:

$$\text{Total Ch} - \left(\frac{\text{(Triglycerides}}{5} + \text{HDL-Ch}\right)$$

(Rifkind, 1970). The atherogenic index (AI) was defined according to Klimov and coworkers as

$$\frac{\text{Total Ch} - \text{HDL-Ch}}{\text{HDL-Ch}}$$

(Suchkova *et al.*, 1978). The triglyceride level of VLDL (VLDL-TG) was found from

$$\text{TG} - \frac{\text{Total Ch}}{5} + \frac{\text{HDL-Ch}}{8}$$

(Golubev, 1981). The statistical treatment of results was on the basis of Student's $t$ test.

### RESULTS

The data presented in Table I show an age-associated rise in the blood levels of total cholesterol, triglycerides, and total

TABLE I.—*Blood-lipid levels (mean ± s.e.) in tumour patients and healthy controls*

| Group | Age (years) | n | Total cholesterol (mg/100 ml) | Triglycerides (mg/100 ml) | Total $\beta$-lipoproteins (units of extinction) | HDL-cholesterol (mg/100 ml) |
|---|---|---|---|---|---|---|
| Control | | | | | | |
| Female | 20–29 | 45 | 192 ± 4 | 88 ± 6 | 388 ± 14 | — |
| | 45–78 | 45 | 246 ± 8 | 103 ± 6 | 459 ± 20 | 59 ± 2 (28) |
| Male | 20–29 | 45 | 184 ± 6 | 109 ± 4 | 382 ± 17 | — |
| | 45–70 | 35 | 241 ± 5 | 139 ± 9 | 538 ± 24 | 53 ± 3 (14) |
| Breast cancer† | | | | | | |
| A | 53 ± 1 | 48 | 251 ± 7 | 156 ± 10* | 586 ± 15* | 51 ± 1* |
| B | 52 ± 0·7 | 137 | 242 ± 3 | 165 ± 7* (58)‡ | 654 ± 16* (75) | — |
| C | 56 ± 0·6 | 73 | 261 ± 6 | 161 ± 6* (69) | 574 ± 20* (63) | 57 ± 3 (21) |
| Lung cancer | 58 ± 0·7 | 113 | 214 ± 4* | 163 ± 6* (77) | 552 ± 12 (59) | 45 ± 2* (15) |
| Cancer of rectum | | | | | | |
| Female | 56 ± 2 | 24 | 243 ± 6 | 149 ± 11* (23) | 580 ± 21* | 56 ± 3 (14) |
| Male | 55 ± 2 | 18 | 253 ± 8 | 152 ± 12 | 566 ± 29 | 48 ± 3·5 (12) |
| Cancer of stomach | | | | | | |
| Female | 52 ± 2 | 20 | 255 ± 15 | 152 ± 15* | 581 ± 29* | 51 ± 3* (9) |
| Male | 52 ± 2 | 22 | 227 ± 9 | 138 ± 8 (21) | 542 ± 28 | 49 ± 3·5 (13) |

* Difference from age- and sex-matched controls is statistically significant ($P < 0·05$).

† Subgroup A—patients with primary tumours in whom blood level of HDL-cholesterol was determined; Subgroup B—primary patients in whom this parameter was not determined; Subgroup C—patients in remission.

‡ Figures in parentheses denote the number of cases, where this differs from the total.

$\beta$-lipoproteins in healthy males and females. The blood concentrations of triglycerides and total $\beta$-lipoproteins in the older group of healthy males were significantly higher than in corresponding group of healthy females ($P < 0·05$) whilst HDL-cholesterol levels were lower ($P < 0·1$). The tumour patients (except males with stomach and rectal tumours) revealed a significantly raised content of triglycerides and total $\beta$-lipoproteins as compared with healthy controls of the same age. In contrast, HDL-cholesterol levels in patients with primary breast, lung, and stomach (females) tumours proved to be significantly lower than in healthy controls of the same sex. The serum total cholesterol concentration in lung-cancer patients was lower than in the healthy males of the older age group; for tumours at other sites it did not show significant differences from age-matched controls. This parameter in breast cancer patients was significantly higher ($P < 0·05$) than in those with fibroadenomatosis, in whom blood cholesterol levels were 217 ± 6 (whole group, n = 59) and 228 ± 10 mg/100 ml (patients over 50 years, n = 22) respectively. It should be stressed that no significant differences in the indices of lipidaemia between primary-breast-cancer patients and those in clinical remission were found (Table I).

It is evident from Table II that the clinical stage of tumour progression had no definite effect on blood-lipid levels (except rectal-cancer patients). VLDL-triglyceride concentration was significantly higher than in controls (Table III). There were no significant differences in the atherogenic index, HDL-cholesterol: total-cholesterol ratio or the LDL-cholesterol concentration in the blood of tumour patients and controls.

The incidence of hyperlipoproteinaemia in healthy controls was lower than in tumour patients (Table IV). Type IIa was more frequent in female controls, and Type IV in males. The latter phenomenon appeared to be more pronounced in male patients with lung cancer, unlike those suffering from rectal and stomach tumours, in whom Type IIa hyperlipoproteinaemia was as frequent as Type IV. A considerably increased incidence of Type IV hyperlipoproteinaemia, and a decreased inci-

TABLE II.—*Blood-lipid levels (mean ± s.e.) in relation to stage of malignant disease*

| Group | Stage | n | Total cholesterol (mg/100 ml) | Triglycerides (mg/100 ml) | Total β-lipoproteins (units of extinction) | HDL-cholesterol (mg/100 ml) |
|---|---|---|---|---|---|---|
| Breast cancer† | I | 11 | 252 ± 17 | 151 ± 12 | 557 ± 37 | 53 ± 3 |
| | II | 11 | 259 ± 15 | 179 ± 15 | 618 ± 29 | 51 ± 3 |
| | III | 22 | 247 ± 11 | 153 ± 17 | 578 ± 18 | 51 ± 2 |
| Lung cancer | I–II | 23 | 210 ± 9 | 171 ± 18 | 533 ± 33 | — |
| | III | 28 | 220 ± 9 | 122 ± 9* | 548 ± 29 | — |
| | IV | 11 | 221 ± 6 | 161 ± 20 | 481 ± 21 | — |
| Cancer of rectum | I–II | 6 | 286 ± 10 | 171 ± 33 | 670 ± 26 | 57 ± 6 |
| | III | 18 | 243 ± 8 | 157 ± 13 | 556 ± 21* | 51 ± 3 |
| | IV | 9 | 233 ± 11* | 119 ± 16 | 505 ± 23* | 54 ± 8 |
| Cancer of stomach | I–II | 9 | 228 ± 14 | 128 ± 17 | 557 ± 32 | 50 ± 3 |
| | III | 20 | 245 ± 12 | 147 ± 12 | 558 ± 31 | 54 ± 4 |
| | IV | 10 | 216 ± 23 | 157 ± 22 | 519 ± 57 | 51 ± 3 |

*Difference from data in patients in Stage I–II is statistically significant.
† Subgroup A in Table I.

TABLE III.—*Some calculated indices of lipidaemia in tumour patients*

| Group | | HDL-Ch/Ch | LDL-Ch | VLDL-TG | AI |
|---|---|---|---|---|---|
| Control (> 45 yr) | Female | 0·22 ± 0·01 | 176 ± 10 | 71 ± 6 | 3·62 ± 0·19 |
| | Male | 0·23 ± 0·02 | 158 ± 6 | 76 ± 17 | 3·65 ± 0·31 |
| Breast cancer | Subgr. A | 0·21 ± 0·01 | 169 ± 5 | 118 ± 7* | 4·08 ± 0·16 |
| | Subgr. C | 0·21 ± 0·01 | 196 ± 9 | 85 ± 9 | 4·16 ± 0·31 |
| Lung cancer | | 0·20 ± 0·01 | 147 ± 9 | 135 ± 12* | 4·42 ± 0·34 |
| Cancer of rectum | Female | 0·24 ± 0·02 | 156 ± 9 | 109 ± 12* | 3·40 ± 0·29 |
| | Male | 0·19 ± 0·01 | 169 ± 8 | 109 ± 15 | 4·31 ± 0·29 |
| Cancer of stomach | Female | 0·23 ± 0·02 | 149 ± 11 | 129 ± 14* | 3·68 ± 0·37 |
| | Male | 0·22 ± 0·01 | 146 ± 9 | 106 ± 8 | 3·97 ± 0·42 |

* Difference from sex-matched controls is statistically significant ($P < 0·05$).
† See Materials and Methods.

TABLE IV.—*Incidence of different types of hyperlipoproteinaemia (%) in tumour patients and healthy controls (based on criteria of Carlson* et al., *1977)*

| Group | | Types of hyperlipoproteinaemia (%) IIa | IIb | IV | Normal |
|---|---|---|---|---|---|
| Control (> 45 yr) | F | 18·7 | 0 | 3·1 | 78·2 |
| | M | 5·7 | 0 | 11·4 | 82·9 |
| Breast cancer | A | 7·5 | 11·7 | 20·2 | 60·6 |
| | C | 21·3 | 9·3 | 20·0 | 49·4 |
| Lung cancer | | 1·3 | 1·3 | 24·7 | 72·7 |
| Cancer of rectum | F | 4·3 | 8·6 | 17·2 | 69·9 |
| | M | 16·7 | 0 | 22·2 | 61·1 |
| Cancer of stomach | F | 11·1 | 16·7 | 11·1 | 61·1 |
| | M | 14·3 | 0 | 19·0 | 66·7 |

dence of Type IIa (primary breast cancer and rectal cancer), when compared with healthy females, proved to be typical of female tumour patients.

### DISCUSSION

The importance of studying lipid metabolism in tumour patients follows from data on the role of cholesterol in cell proliferation (H. W. Chen *et al.*, 1977), on the inhibitory effects of disturbances of carbohydrate–lipid metabolism on the immune system (Dilman, 1977, 1978*b*), on the immunosuppressive effects of some particular lipids, like polyunsaturated fatty acids (Meade & Mertin, 1978), on the possibility of influencing tumour growth by changes in lipid metabolism (Littman *et al.*, 1966).

Questions concerning lipid metabolism cannot be discussed without taking into consideration the fact that the blood lipids are components of circulating lipoproteins. Data on the total cholesterol and triglyceride and on HDL-cholesterol content of blood of tumour patients, presented in Table I, allow one to draw some conclusions about lipoprotein concentration, after appropriate calculations (see Methods) have been made. It should be mentioned that any calculations of this kind are valid provided there are no significant differences in lipoprotein composition between controls and cancer patients. We found no evidence in the literature for such differences. The data in Table III suggest that the main reason for the apparent changes of cholesterol and triglyceride concentrations in the blood of the patients examined is the rise of VLDL, the triglyceride carrier. This appears to be the cause of the rise of $\beta$-lipoproteins in tumour patients observed by other investigators (Nanava & Tzinzadze, 1961; Miller & Erf, 1956) as well as in the present study. The concentration of HDL in the blood of some groups of tumour patients, on the other hand, is reduced as assessed by HDL-cholesterol concentration (Table I).

As to the results of typing of hyperlipidaemia, it should be mentioned first that dealing with oncological patients, we exclude those rare primary disorders of lipid metabolism that were the basis for the original concept of different types of hyperlipidaemia. What we are dealing with here should be regarded rather as phenocopies of the corresponding types of hyperlipidaemia.

The prevalence of Type IV hyperlipoproteinaemia in cancer patients (Table IV) might be due to a reduced rate of fractional turnover of VLDL (*i.e.* of triglycerides), which is common in moderate Type IV hyperlipoproteinaemia (Havel *et al.*, 1970; Quarfordt *et al.*, 1970; Olefsky *et al.*, 1974; Rössner *et al.*, 1976) or due to enhanced synthesis of triglycerides. The observation that the concentrations of triglyceride and other lipids in the blood of tumour patients do not depend upon the stage of the disease (Table II) militates against the concept that the tumour itself plays the primary role in the disturbances of lipid metabolism (Begg, 1958; Liebelt *et al.*, 1971) and does not support the idea that a steady decrease in blood lipid concentrations during tumour progression reflects the dubious prognosis for the patients (Rose *et al.*, 1974; Chao *et al.*, 1975).

Another argument against the primary role of the tumour in disturbing the lipid metabolism of tumour patients is the persistence of hyperlipidaemia in breast-cancer patients for months and even years after radical mastectomy (Table I). The above evidence suggests that high blood lipid levels in tumour patients reflects a higher risk for subjects with hyperlipidaemia of developing tumours, rather than the presence of a tumour. This conclusion is supported by the findings of Barclay & Skipsky (1975) that the level of HDL is diminished and of VLDL is raised not only in cancer patients, but also in healthy subjects from families with a cancer history. Besides, the VLDL and LDL levels rise in the course of normal ageing (see Table I) as a result of the primary hypothalamic shifts. This point is discussed in detail in Dilman (1968, 1979, 1980) and Dilman *et al.* (1979). It should be stressed that from this point of view only the levels of lipids and other metabolites characteristic for the ages of 20–25 years, when cancer incidence is lowest, may be considered as normal, so even when there is no difference in blood lipids between cancer patients and age-matched controls, the patients should be regarded as hyperlipidaemic compared with healthy persons aged 20–25 (Dilman, 1979, 1980).

Such an approach treats metabolic disorders which are characteristic of cancer as components of the syndrome of cancerophilia. This term needs comment. The concept of predisposition to cancer is widespread. But anyone using it has his own opinion of what it should mean. In our opinion no matter what the immediate causes of malignant transformation, there are 3 main factors promoting this process: (1) enhanced cell proliferation; (2) reduced cellular immunity; and (3) reduced DNA repair. There is evidence that hyperlipidaemia of the type that develops in the course of normal ageing (characterized by lowered glucose tolerance, by reactive hyperinsulinaemia and by other related metabolic disturbances) may promote all 3 of them. For example, hyperlipidaemic serum enhances cell proliferation (R. M. Chen *et al.*, 1977). Hyperlipidaemia causes metabolic immunodepression (Dilman, 1977, 1978*a*,*b*). Preliminary data from our laboratory demonstrate a negative correlation between the cholesterol content of blood and lymphocytes on the one hand, and the rate of DNA repair on the other. The complex of metabolic disorders that promotes the development of the above 3 conditions favourable for tumour progression has been designated as cancerophilia (Dilman, 1977, 1978*a*,*b*). This complex must include not only metabolic but also related hormonal shifts. In particular, hyperinsulinaemia, often connected with hypertriglyceridaemia, should be mentioned as a factor promoting cell proliferation.

It should be borne in mind that the systemic effects of a tumour (Shapot, 1975) may further aggravate metabolic disorders that served as a risk factor for the appearance of the tumour, and so may cause secondary cancerophilia. The evidence presented in this paper suggests the possibility of using dietary and pharmacological means of correction of lipid metabolism in cancer prophylaxis and therapy. The evaluation of such treatment of cancer is under investigation.

In a recent editorial in *The Lancet* (1980) it was stated that a lowered cholesterol level predisposes to cancer. The dependence of cancer incidence upon blood cholesterol may be rather complicated. The data of Rose & Shipley (1980) show a connection between lowered blood cholesterol and the appearance of colonic cancer 2 years later. When the interval between examination of blood cholesterol and the appearance of a tumour was more than 2 years, there was a positive correlation between blood cholesterol level and cancer incidence. In the former case the lowered cholesterol level could be a result of systemic effects of the tumour which does not manifest itself clinically. In the latter case high cholesterol level could be a true risk factor for tumour development. Besides this, the data of Westlung & Nicolaysen (1972) showed that extremely high levels of blood cholesterol may be connected with a lower cancer incidence. There may be special reasons (*e.g.* genetic disorders) for these high levels which may be unrelated to lipid abnormalities found in cancerophilia.

### REFERENCES

BARCLAY, M. & SKIPSKY, V. (1975) Lipoproteins in relation to cancer. *Prog. Biochem. Pharmacol.*, **10**, 76.

BARCLAY, M., SKIPSKY, V., TEREBUS-KEKISH, O., GREENS, E. M., KAUFMAN, R. J. & STOCK, C. C. (1970) Effect of cancer upon high-density and other lipoproteins. *Cancer Res.*, **30**, 2420.

BEGG, R. W. (1958) Tumor–host relation *Adv. Cancer Res.*, **5**, 1.

BERSTEIN, L. M., VALDINA, E. A., BARCHUK, A. S., FADEJEV, N. P. & LVOVICH, E. G. (1978) Adaptation and thyroid homoeostasis and the state of fat-carbohydrate metabolism in lung cancer patients. *Voprosi Onkol.*, **24** (2), 48 (in Russian).

BURSTEIN, M. & SCHOLNICK, N. R. (1973) Lipoprotein polyanions metal interaction. *Adv. Lipid Res.*, **11**, 68.

CARLSON, L. A. (1963) Determination of serum triglycerides. *Atheroscl. Res.*, **3**, 334.

CARLSON, L. A., DANIELSON, M., EKBERG, J., KLINTEMAR, B. & ROSENHAMER, G. (1977) Reduction of myocardial reinfarction by the combined treatment with clofibrate and nicotinic acid. *Atherosclerosis*, **28**, 81.

CHAO, F.-C., EFRON, B. & WOLF, P. (1975) The possible prognostic usefulness of assessing serum proteins and cholesterol in malignancy. *Cancer*, **35**, 1223.

CHEN, H. W., KANDUTSCH, A. A. & HEINIGER, H. J. (1977) The role of cholesterol in malignancy. *Prog. Exp. Tumor Res.* (Basel), **22**, 275.

CHEN, R. M., GETZ, G. S., FISHER-DZOGA, K. & WISSLER, R. W. (1977) The role of hyperlipidemic serum in the proliferation and necrosis of aortic medial cells *in vitro*. *Exp. Mol. Pathol.*, **26**, 359.

DILMAN, V. M. (1968) *Ageing, Climax and Cancer.* Leningrad: Medizina (in Russian).

DILMAN, V. M. (1977) Metabolic immunodepression which increases the risk of cancer. *Lancet*, ii, 1207.

DILMAN, V. M. (1978*a*) Transformation of programme of development into the mechanism of age pathology. Elevation model of age pathology and natural death of humans. *Human Physiol.*, **4**, 579 (in Russian).

DILMAN, V. M. (1978*b*) Ageing, metabolic immunodepression and carcinogenesis. *Mech. Ageing Dev.*, **8**, 153.

DILMAN, V. M. (1979) Hypothalamic mechanisms of ageing and of specific age pathology and natural death. *Exp. Gerontol.*, **14**, 287.

DILMAN, V. M. (1980) *The Law of Deviation of Homeostasis and Diseases of Aging.* Littleton, Mass.: PSG Publ.

DILMAN, V. M., BERSTEIN, L. M., BOBROV, Y. F., BOCHMAN, J. V., KOVALEVA, I. G. & KRYLOVA, N. V. (1968) Hypothalamo-pituitary hyperactivity and endometrial carcinoma. *Am. J. Obstet. Gynecol.*, **102**, 880.

DILMAN, V. M. & BOBROV, Y. F. (1966) Hypercholesterolemia and cancer. In *Modern Problems of Oncology.* Leningrad: Medizina. p. 76 (in Russian).

DILMAN, V. M., LAPIN, I. P. & OXENKRUG, G. F. (1979) Serotonin and aging. In *Serotonin in Health and Disease.* Vol. 5. Ed. Essman. New York: Spectrum. p. 111.

EDITORIAL (1980) The link between cholesterol and cancer. *Lancet*, ii, 243.

EISENBERG, S. & LEVY, R. J. (1975) Lipoprotein metabolism. *Adv. Lipid Res.*, **13**, 1.

FELDMAN, E. & CARTER, A. C. (1971) Circulation lipid and lipoproteins in women with metastatic breast carcinoma. *J. Clin. Endocrinol. Metab.*, **33**, 8.

GOLUBEV, A. G. (1981) Use of calculations for estimation of blood lipoprotein concentrations on the basis of total triglycerides and cholesterol levels (in press).

HAVEL, R. J., KANE, J. R., BALASSE, E. O., SEGAL, N. & BASSO, L. V. (1970) Splanchnic metabolism of free fatty acids and production of triglycerides of very low density lipoproteins in normotriglyceridemia and hypertriglyceridemic subjects. *J. Clin. Invest.*, **49**, 2017.

KAVETSKY, R. E. (1962) *Tumor and Host.* Kiev: Naukova Dumka (in Russian).

KING, E. G. (1947) Cholesterol (Method of Sackett). In *Microanalysis in Medical Biochemistry.* London: Churchill. p. 16.

LEDVINA, M. & COUFALOVA, S. (1960) The serum β-lipoprotein level in tumors. *Neoplasma*, **7**, 419.

LIEBELT, R. A., LIEBELT, A. G. & JOHNSTON, H. M. (1971) Lipid mobilization and food intake in experimentally obese mice bearing transplantable tumors. *Proc. Soc. Exp. Biol. Med.*, **138**, 482.

LITTMAN, M. L., TAGUCHI, T. & MOSBACH, E. H. (1966) Effect of cholesterol-free, fat-free diet and hypocholesteremic agents on growth of transplantable animal tumors. *Cancer Chemother. Rep.*, **50**, 25.

MEADE, C. J. & MERTIN, J. (1978) Fatty acids and immunity. *Adv. Lipid Res.*, **16**, 127.

MILLER, B. & ERF, L. (1956) The serum proteins and lipoproteins in patients with carcinoma and subjects free of recurrence. *Surg. Gynecol. Obstet.*, **102**, 487.

NANAVA, J. G. & TSINZADZE, T. M. (1961) Changes in serum lipoproteins spectrum in breast cancer patients. *Scientific Papers of Georgian Oncol. Inst.* (*Tbilizi, USSR*), **139** (in Russian).

OLEFSKY, J., FARQUHAR, J. & REAVEN, G. M. (1974) Sex differences in the kinetics of triglyceride metabolism in normal and hypertriglyceridemic human subjects. *Eur. J. Clin. Invest.*, **4**, 121.

QUARFORDT, S. H., FRANK, A., SHAMES, D. M., BERMAN, M. & STEINBERG, D. (1970) Very low density lipoprotein triglyceride transport in Type IV hyperlipoproteinemia and the effect of carbohydrate-rich diet. *J. Clin. Invest.*, **49**, 2281.

RIFKIND, B. (1970) Typing of hyperlipoproteinemia. *Atherosclerosis*, **11**, 545.

ROSE, G., BLACKBURN, H., KEYS, A. & 5 others (1974) Colon cancer and blood cholesterol. *Lancet*, i, 181.

ROSE, G. & SHIPLEY, M. (1980) Plasma lipids and mortality: A source of error. *Lancet*, i, 523.

RÖSSNER, S., EKLUND, B., KAISER, U., OLSSON, A. G. & WALLDINGS, G. (1976) Removal of exogenous plasma triglycerides in forearm muscle and subcutaneous tissue of normo- and hypertriglyceridemic man. *Eur. J. Clin. Invest.*, **6**, 259.

SHAPOT, V. S. (1975) *Biochemical problems of tumor growth.* Moscow: Medizina (in Russian).

SMETHURST, M., BASU, T. K. & WILLIAMS, D. C. (1975) Levels of cholesterol, 11-oxycorticosteroids and progesterone in plasma from menopausal women with breast cancer. *Eur. J. Cancer*, **11**, 751.

SUCHKOVA, S. N., TITOV, V. N., KURCHATOVA, S. A. & KLIMOV, A. N. (1978) New approaches to the evaluation of cholesterol fractions of plasma lipoproteins in normo- and hyperlipidemias. *Kardiologia*, **6**, 29 (in Russian).

TSYRLINA, E. V., BERSTEIN, L. M., SEMIGLASOV, V. F., VASILIEVA, I. A., KHOLDIN, S. A. & DILMAN, V. M. (1977) Endocrine-metabolic criteria for distinguishing two pathogenetic types of breast cancer. *Neoplasma*, **24**, 199.

WESTLUNG, K. & NICOLAYSEN, R. (1972) Ten years mortality and morbidity related to serum cholesterol level. A follow-up of 3751 men aged 40–49. *Scand. J. Clin. Lab. Invest.*, **30** (Suppl. 127), 1.


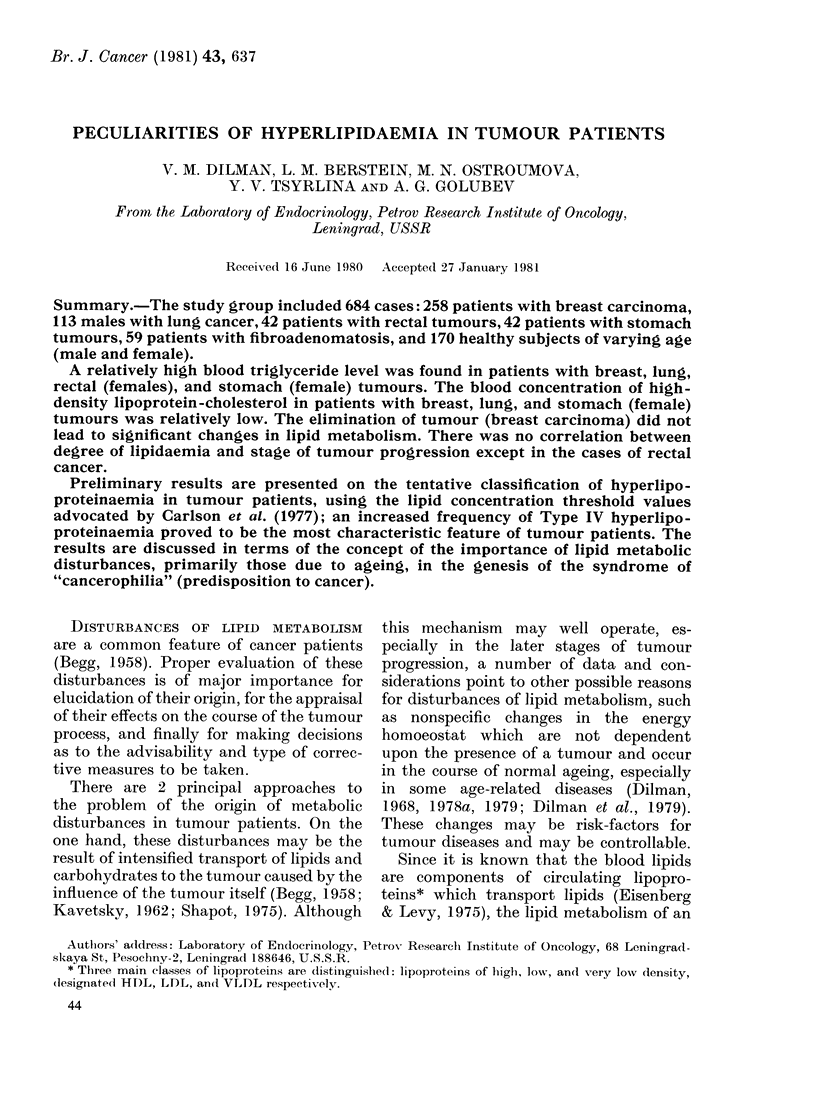

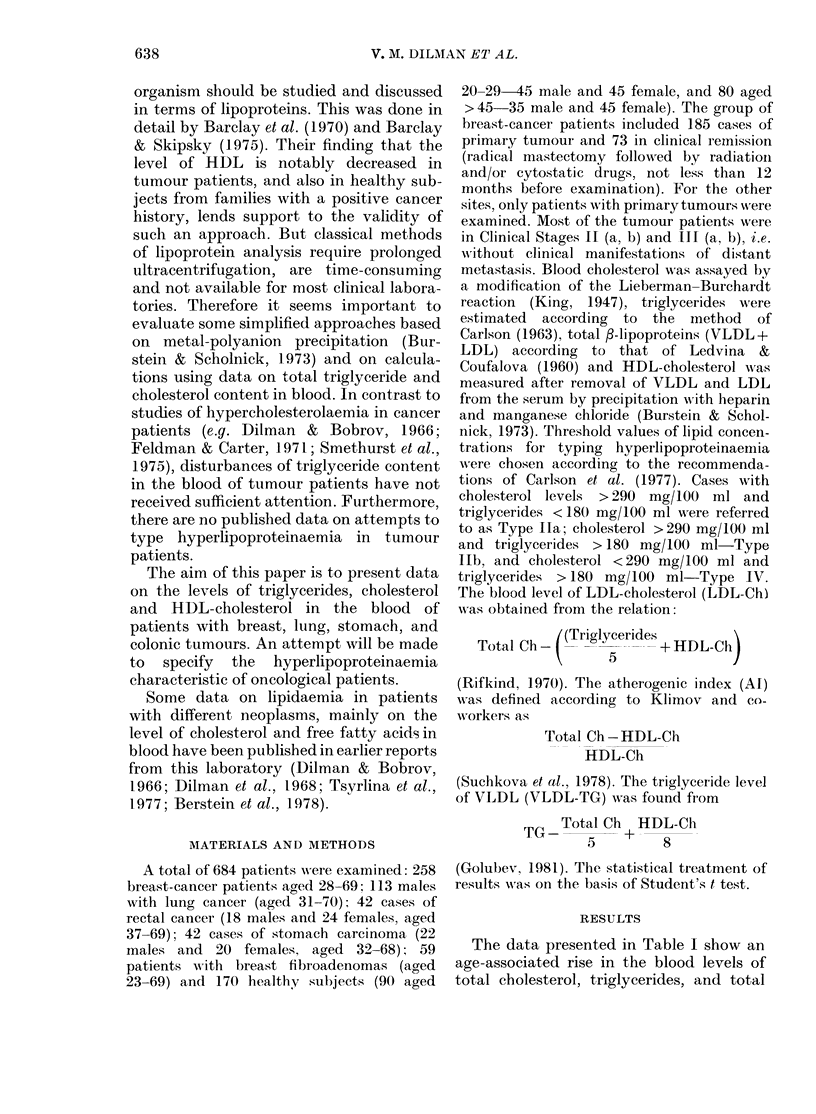

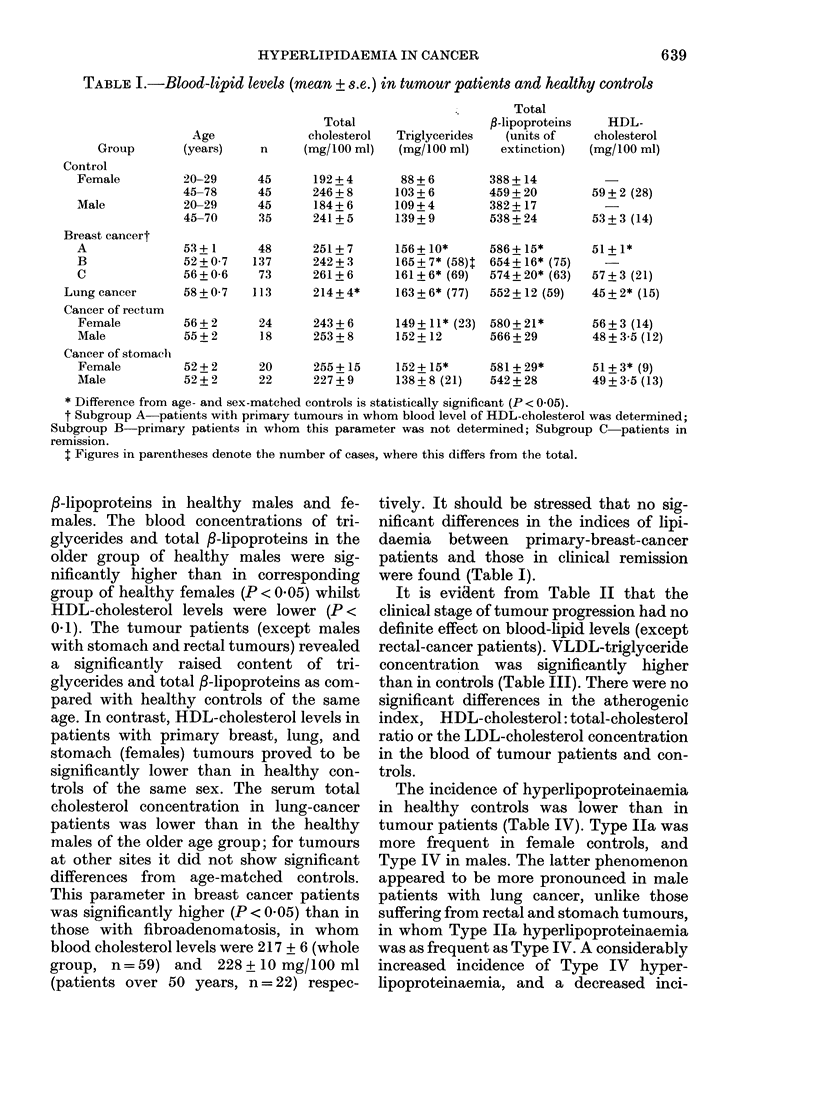

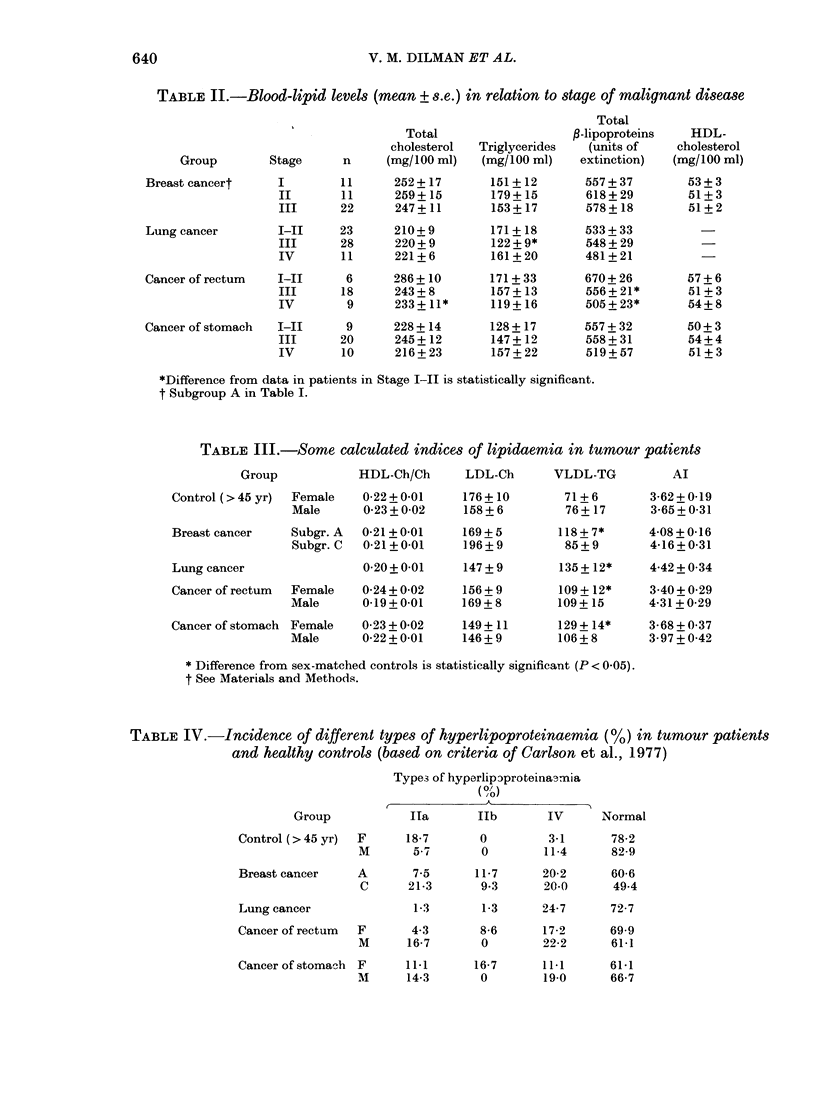

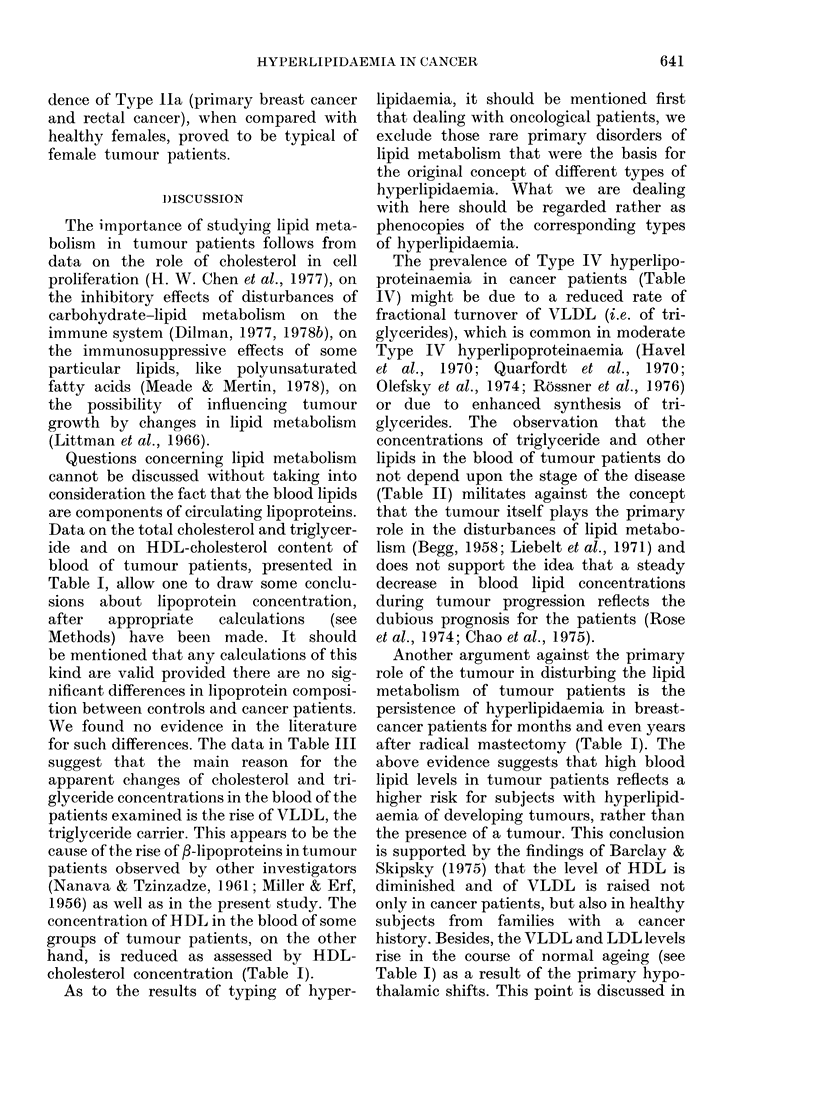

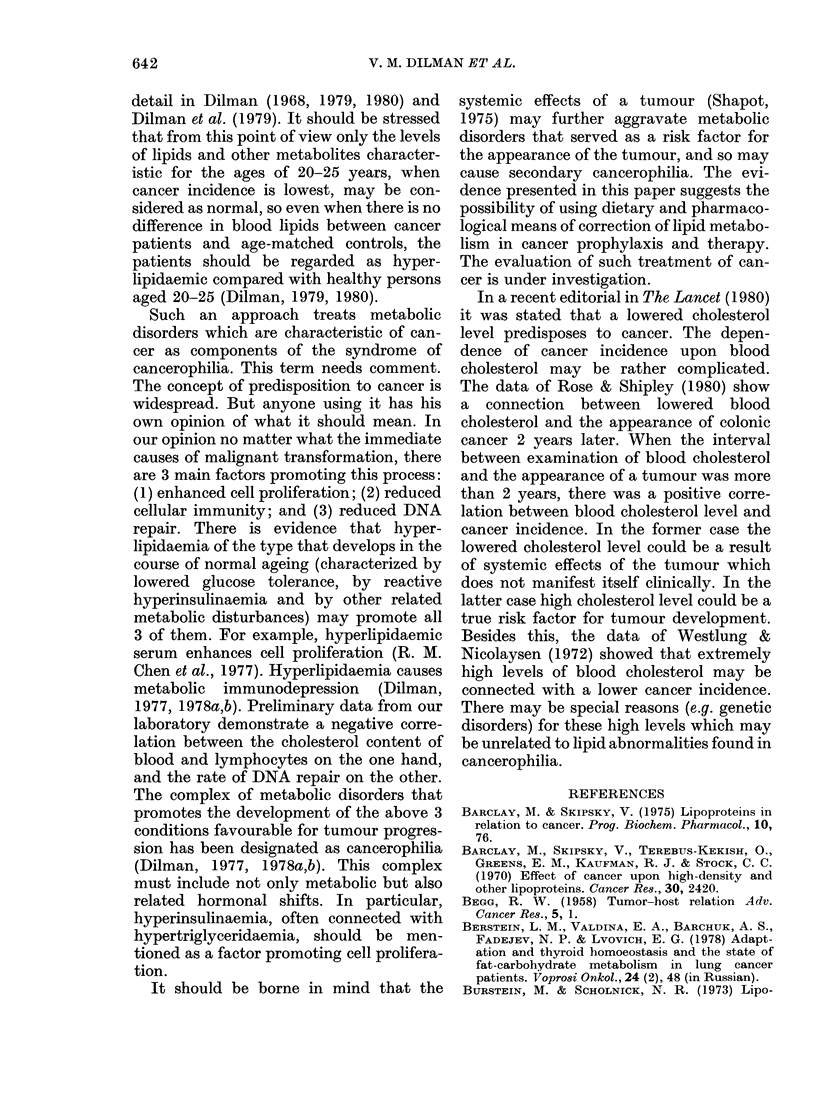

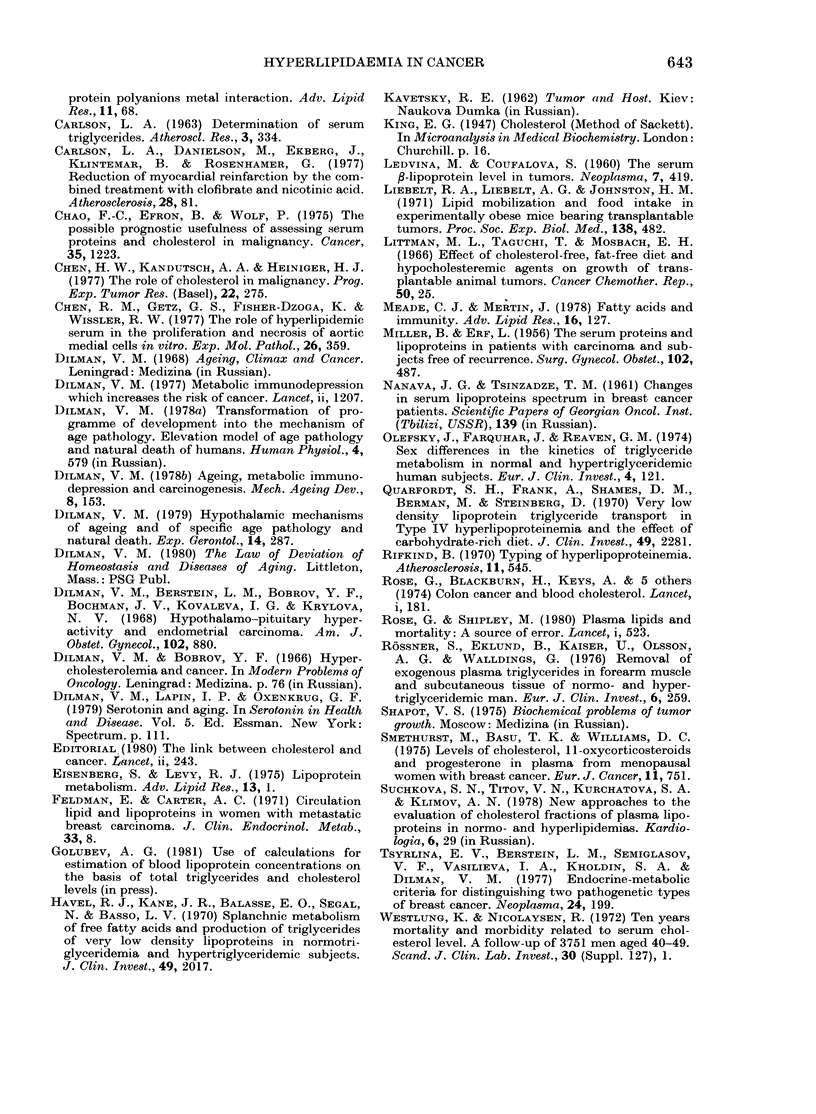


## References

[OCR_00688] Barclay M., Skipski V. P. (1975). Lipoproteins in relation to cancer.. Prog Biochem Pharmacol.

[OCR_00693] Barclay M., Skipski V. P., Terebus-Kekish O., Greene E. M., Kaufman R. J., Stock C. C. (1970). Effects of cancer upon high-density and other lipoproteins.. Cancer Res.

[OCR_00717] CARLSON L. A. (1963). DETERMINATION OF SERUM TRIGLYCERIDES.. J Atheroscler Res.

[OCR_00721] Carlson L. A., Danielson M., Ekberg I., Klintemar B., Rosenhamer G. (1977). Reduction of myocardial reinfarction by the combined treatment with clofibrate and nicotinic acid.. Atherosclerosis.

[OCR_00728] Chao F. C., Efron B., Wolf P. (1975). The possible prognostic usefulness of assessing serum proteins and cholesterol in malignancy.. Cancer.

[OCR_00734] Chen H. W., Kandutsch A. A., Heiniger H. J. (1978). The role of cholesterol in malignancy.. Prog Exp Tumor Res.

[OCR_00739] Chen R. M., Getz G. S., Fischer-Dzoga K., Wissler R. W. (1977). The role of hyperlipidemic serum on the proliferation and necrosis of aortic medial cells in vitro.. Exp Mol Pathol.

[OCR_00759] Dilman V. M. (1978). Ageing, metabolic immunodepression and carcinogenesis.. Mech Ageing Dev.

[OCR_00774] Dilman V. M., Berstein L. M., Bobrov Y. F., Bohman Y. V., Kovaleva I. G., Krylova N. V. (1968). Hypothalamopituitary hyperactivity and endometrial carcinoma. Qualitative and quantitative disturbances in hormone production.. Am J Obstet Gynecol.

[OCR_00764] Dilman V. M. (1979). Hypothalamic mechanisms of ageing and of specific age pathology--V. A model for the mechanism of human specific age pathology and natural death.. Exp Gerontol.

[OCR_00749] Dilman V. M. (1977). Metabolic immunodepression which increases the risk of cancer.. Lancet.

[OCR_00795] Eisenberg S., Levy R. I. (1975). Lipoprotein metabolism.. Adv Lipid Res.

[OCR_00799] Feldman E. B., Carter A. C. (1971). Circulating lipids and lipoproteins in women with metastatic breast carcinoma.. J Clin Endocrinol Metab.

[OCR_00811] Havel R. J., Kane J. P., Balasse E. O., Segel N., Basso L. V. (1970). Splanchnic metabolism of free fatty acids and production of triglycerides of very low density lipoproteins in normotriglyceridemic and hypertriglyceridemic humans.. J Clin Invest.

[OCR_00828] LEDVINA M., COUFALOVA S. (1960). The serum beta-lipoprotein level in tumours.. Neoplasma.

[OCR_00831] Liebelt R. A., Liebelt A. G., Johnston H. M. (1971). Lipid mobilization and food intake in experimentally obese mice bearing transplanted tumors.. Proc Soc Exp Biol Med.

[OCR_00837] Littman M. L., Taguchi T., Mosbach E. H. (1966). Effect of cholesterol-free, fat-free diet and hypocholesteremic agents on growth of transplantable animal tumors.. Cancer Chemother Rep.

[OCR_00848] MILLER B. J., ERF L. (1956). The serum proteins and lipoproteins in patients with carcinoma and in subjects free of recurrence.. Surg Gynecol Obstet.

[OCR_00844] Meade C. J., Mertin J. (1978). Fatty acids and immunity.. Adv Lipid Res.

[OCR_00860] Olefsky J., Farquhar J. W., Reaven G. M. (1974). Sex difference in the kinetics of triglyceride metabolism in normal and hypertriglyceridaemic human subjects.. Eur J Clin Invest.

[OCR_00866] Quarfordt S. H., Frank A., Shames D. M., Berman M., Steinberg D. (1970). Very low density lipoprotein triglyceride transport in type IV hyperlipoproteinemia and the effects of carbohydrate-rich diets.. J Clin Invest.

[OCR_00872] Rifkind B. (1970). Typing of hyperlipoproteinaemias.. Atherosclerosis.

[OCR_00876] Rose G., Blackburn H., Keys A., Taylor H. L., Kannel W. B., Paul O., Reid D. D., Stamler J. (1974). Colon cancer and blood-cholesterol.. Lancet.

[OCR_00881] Rose G., Shipley M. J. (1980). Plasma lipids and mortality: a source of error.. Lancet.

[OCR_00895] Smethurst M., Basu T. K., Williams D. C. (1975). Levels of cholesterol, 11-hydroxycorticosteroids and progesterone in plasma from postmenopausal women with breast cancer.. Eur J Cancer.

[OCR_00907] Tsyrlina E. V., Bershtein L. M., Semiglazov V. F., Vasilieva I. A., Kholdin S. A., Dilman V. M. (1977). Endocrine-metabolic criteria for distinguishing two pathogenetic types of breast cancer.. Neoplasma.

[OCR_00914] Westlund K., Nicolaysen R. (1972). Ten-year mortality and morbidity related to serum cholesterol. A follow-up of 3.751 men aged 40-49.. Scand J Clin Lab Invest Suppl.

